# Nationwide evaluation of mutation-tailored treatment of gastrointestinal stromal tumors in daily clinical practice

**DOI:** 10.1007/s10120-021-01190-9

**Published:** 2021-04-28

**Authors:** Elisabeth M. P. Steeghs, Hans Gelderblom, Vincent K. Y. Ho, Quirinus J. M. Voorham, Stefan M. Willems, PATH consortium, Katrien Grünberg, Marjolijn J. L. Ligtenberg

**Affiliations:** 1 Department of Pathology, Radboud University Medical Center, Nijmegen, The Netherlands; 2 Department of Medical Oncology, Leiden University Medical Center, Leiden, The Netherlands; 3 Departments of Research and Development, Netherlands Comprehensive Cancer Organization (IKNL), Utrecht, The Netherlands; 4 PALGA Foundation, Houten, The Netherlands; 5 Department of Pathology and Medical Biology, University Medical Center Groningen, Groningen, The Netherlands; 6 Laboratory of Tumor Genetics, Department of Pathology and Human Genetics, Radboud University Medical Center, Geert Grooteplein Zuid 10, PO Box 9101, 6500 HB Nijmegen, The Netherlands

**Keywords:** Gastrointestinal stromal tumor, Predictive genetic testing, Imatinib, Molecular targeted therapy, Guidelines, KIT, PDGFRA

## Abstract

**Background:**

Molecular analysis of *KIT* and *PDGFRA* is critical for tyrosine kinase inhibitor treatment selection of gastrointestinal stromal tumors (GISTs) and hence recommended by international guidelines. We performed a nationwide study into the application of predictive mutation testing in GIST patients and its impact on targeted treatment decisions in clinical practice.

**Methods:**

Real-world clinical and pathology information was obtained from GIST patients with initial diagnosis in 2017–2018 through database linkage between the Netherlands Cancer Registry and the nationwide Dutch Pathology Registry.

**Results:**

Predictive mutation analysis was performed in 89% of the patients with high risk or metastatic disease. Molecular testing rates were higher for patients treated in expertise centers (96%) compared to non-expertise centers (75%, *P* < 0.01). Imatinib therapy was applied in 81% of the patients with high risk or metastatic disease without patient’s refusal or adverse characteristics, e.g., comorbidities or resistance mutations. Mutation analysis that was performed in 97% of these imatinib-treated cases, did not guarantee mutation-tailored treatment: 2% of these patients had the *PDGFRA* p.D842V resistance mutation and 7% initiated imatinib therapy at the normal instead of high dose despite of having a *KIT* exon 9 mutation.

**Conclusion:**

In conclusion, nationwide real-world data show that over 81% of the eligible high risk or metastatic disease patients receive targeted therapy, which was tailored to the mutation status as recommended in guidelines in 88% of cases. Therefore, still 27% of these GIST patients misses out on mutation-tailored treatment. The reasons for suboptimal uptake of testing and treatment require further study.

**Supplementary Information:**

The online version contains supplementary material available at 10.1007/s10120-021-01190-9.

## Introduction

Gastrointestinal stromal tumors (GISTs) are the most common primary mesenchymal neoplasms of the gastrointestinal tract. The majority of GISTs (75–80%) have one or more somatic mutations in the proto-oncogene *KIT* [1, 2]. These mainly affect the juxtamembrane domain (encoded by exon 11), followed by mutations in the extracellular domain of KIT (encoded by exon 9). Primary mutations in the intracellular ATP-binding region and activation loop of the kinase domain of KIT (exon 13 and 17, respectively) are observed in a low percentage of tumors. In *KIT*-negative GISTs, activating somatic mutations in *PDGFRA* are found in 20–25% of cases [3, 4], including mutations in the activation loop (exon 18), juxtamembrane domain (exon 12), and ATP-binding domain (exon 14). GISTs without mutations in *KIT* or *PDGFRA* are a heterogeneous group that display various oncogenic mutations, including mutations in *BRAF*, succinate dehydrogenase (SDH) subunits genes, *NF1*, or the RAS family [5, 6].

Prognosis varies greatly depending on the malignant potential of the tumor, defined by tumor size, tumor location, the mitotic rate and presence of tumor rupture during surgery [7, 8]. While most GISTs are primarily treated with surgery [9], the tyrosine kinase inhibitor (TKI) imatinib has proven to be effective in prolonging survival of patients with a high risk of recurrence after surgery and cases with locally advanced, unresectable and/or metastatic disease [10–14]. However, sensitivity to imatinib therapy depends on the type of initial *KIT*/*PDGFRA* mutation [15–17]. Imatinib binds to the inactive state of the kinase domains of KIT and PDGFRA, resulting in stabilization of the ‘closed’ conformation. Hence, mutations that favor the active conformation of the kinase domain disfavor imatinib binding. Consequently these patients are less sensitive (*KIT* exon 9) or resistant (*PDGFRA* exon 18 p.D842V) to imatinib and therefore require higher imatinib doses or should be excluded from imatinib therapy, respectively [18–21]. Genetic testing to guide dose selection of imatinib or to selectively withhold imatinib from patients with the *PDGFRA* p.D842V variation was reported to be cost-effective [22, 23]. Thus, targeted therapy in GIST requires both in-depth molecular analysis and interpretation.

Guidelines for molecular analysis and targeted therapy have been developed to assist in the care of patients with GIST [19, 20, 24–26]. Although many of these guidelines were revised several times, only a few studies investigated compliance to guidelines in clinical practice [27–32]. Insight into real-world clinical management of GIST patients may guide further optimization of access to state-of-the-art patient care. In the current study, we used nationwide real-world data to investigate how effectively awareness of predictive mutation analysis has penetrated in routine clinical practice. In addition, we assessed whether molecular test results affected treatment decisions.

## Methods

### Databases and data linkage

**Fig. 1:**
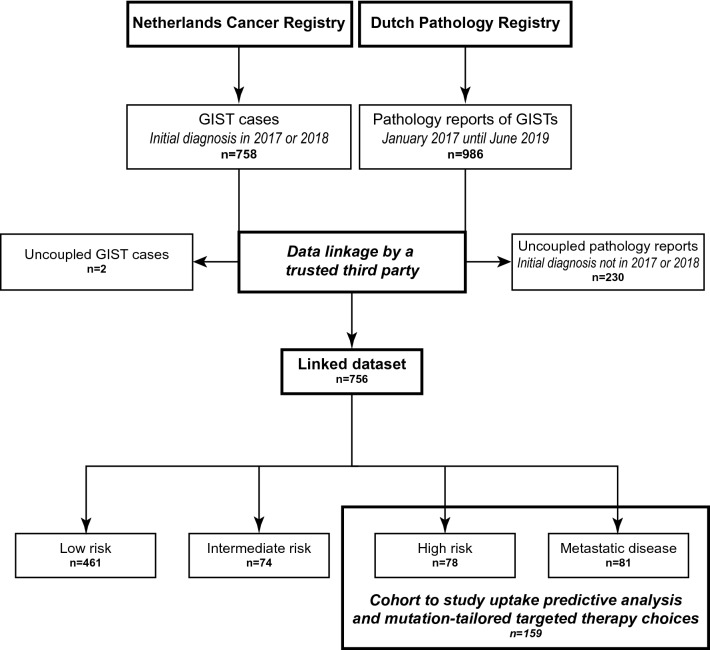
Overview of data collection by the NCR and PALGA. Flow chart of data collection by the NCR and PALGA [33]. Data were linked by a trusted third party, which enabled evaluation of uptake of molecular testing and mutation-informed targeted therapy choice

Clinical and pathology data were obtained from data linkage between the Netherlands Cancer Registry (NCR) and the nationwide network and registry of histo- and cytopathology in the Netherlands (PALGA) [33]. Both databases cover the entire Dutch population (approximately 17.2 million inhabitants). From the NCR, clinical characteristics of patients with a primary GIST diagnosis in 2017 or 2018 were obtained. These variables were registered 6–9 months after initial diagnosis and included: age at initial diagnosis, tumor localization, tumor size, distant metastasis, performance status, surgery, primary therapy details (agent, dose), whether a patient was excluded from further therapy (e.g., due to comorbidities), vital status, time from initial diagnosis to last follow-up date, and whether a patient was evaluated for treatment in an expertise center as defined by Verschoor et al*.* [31] (i.e., five centers with more than 15 new pathology diagnoses of GIST per year in 2011/2012 and a dedicated multidisciplinary sarcoma team). Risk stratification of cases with localized disease was performed according to the AFIP-Miettinen criteria [8] (Supplementary Table 1). Uptake of predictive analysis and imatinib therapy was studied in patients having an established indication for imatinib therapy (i.e., all patients with high risk or metastatic disease). The NCR did not contain information on treatment beyond the first-line, nor on disease progression or recurrence. Via a trusted third party (ZorgTTP [34]), the clinical data were linked to the pathology data (Fig. 1). This linkage was successful for 756/758 GIST patients. Pathology reports were collected from January 2017 to June 2019 using specific queries, which yielded 1977 reports of 986 patients. Manual curation of pathology reports resulted in 545 patients undergoing molecular analyses (Supplementary Fig. 1). Initial diagnosis of 374 of these cases was in 2017 or 2018. 171 cases were diagnosed before 2017 (follow-up samples) or after 2018. Details of the molecular analyses (i.e. technique, gene panel, diagnostic yield) were manually extracted from the reports and annotated. In addition, the pathology department that firstly described the GIST in a report was annotated as department of initial diagnosis. This department could either be located in an expertise center, tertiary cancer center (i.e., academic hospital that is no GIST expertise center), peripheral center with molecular diagnostic facilities, or peripheral center without molecular diagnostic facilities.

**Table 1:** Clinical characteristics of patients diagnosed with GIST in 2017 or 2018

	Total cohort *N* = 758	Uptake molecular testing *N* = 756; 49%^††^			Uptake predictive analysis^†^ *N* = 159; 89%^††^		
	*N (%)*	% (*N*)	P^*^	OR^**^	%	P^*^	OR^**^
Gender			ns			ns	
Male	380 (50.1%)	52.8% (200)			82.3% (90)		
Female	378 (49.9%)	46.2% (174)			92.8% (51)		
Age at initial diagnosis			**ns**			**ns**	
< 50	94 (12.4%)	53.2% (50)			94.1% (16)		
50–70	303 (40.0%)	60.7% (184)			91.5% (65)		
> 70	361 (47.6%)	39% (140)			84.5% (60)		
Risk group at initial diagnosis			** < 0.001**			**ns**	
Low risk	461 (60.8%)	32.8% (151)	** < 0.001**	**0.2**			
Low risk, non-rectal GISTs < 2 cm	*139 (18.3%)*	14.4% *(20)*	** < 0.001**	***0.1***			
Low risk, other	*322 (43.8%)*	40.7% *(131)*	** < 0.001**	***0.5***			
Intermediate risk	74 (9.8%)	67.6% (50)	**0.001**	**2.3**			
High	78 (10.3%)	93.6% (73)	** < 0.001**	**18.2**	93.6% (73)		
Metastatic disease	81 (10.7%)	84% (68)	** < 0.001**	**6.3**	84.0% (68)		
NOS	62 (8.4%)	51.6% (32)	**ns**				
Topography^‡^			** < 0.001**			**ns**	
Colon	9 (1.2%)	33.3% (3)	**ns**		66.7% (2)		
Esophagus	16 (2.1%)	18.8% (3)	**0.034**	**0.2**	100.0% (2)		
Rectum	19 (2.5%)	89.5% (17)	** < 0.001**	**9.0**	62.5% (5)		
Small intestines	177 (23.4%)	60.5% (107)	**0.002**	**1.8**	93.0% (53)		
Stomach	521 (68.7%)	44.3% (230)	** < 0.001**	**0.5**	87.3% (69)		
Peritoneum	5 (0.7%)	100% (5)	**0.029**		100.0% (3)		
Unspecified	16 (2.1%)	56.3% (9)	**ns**		70.0% (7)		
Mitotic rate per 50 high power fields^‡^			** < 0.001**			**ns**	
≤ 5	548 (72.3%)	39.2% (215)	** < 0.001**	**0.2**	80.0% (32)		
> 5	127 (16.8%)	85% (108)	** < 0.001**	**8.1**	93.6% (88)		
Unspecified	83 (10.9%)	63% (51)	**0.013**	**1.9**	84.0% (21)		
Performance status			**0.015**			**ns**	
WHO 0	208 (27.4%)	55.8% (116)	**0.034**	**1.4**	88.9% (40)		
WHO 1	93 (12.3%)	59.1% (55)	**ns**		96.9% (31)		
WHO 2	15 (2.0%)	53.3% (8)	**ns**		80.0% (4)		
WHO 3	7 (0.9%)	57.1% (4)	**ns**		66.7% (2)		
Unspecified	435 (57.4%)	44.1% (191)	**0.001**	**0.6**	86.5% (64)		
Tumor grade			** < 0.001**			**0.011**	
Well differentiated	469 (61.9%)	35.9% (168)	** < 0.001**	**0.2**	75.0% (18)	**0.033**	**0.3**
Moderately differentiated	64 (8.4%)	78.1% (50)	** < 0.001**	**4.0**	96.3% (26)	**ns**	
Poorly differentiated/undifferentiated	83 (10.9%)	92.7% (76)	** < 0.001**	**15.9**	95.4% (62)	**0.039**	**3.9**
Unknown	142 (18.7%)	56.3% (80)	**ns**		81.4% (35)	**ns**	
Surgery			** < 0.001**			**ns**	
Yes	628 (82.8%)	46.6% (292)	** < 0.001**	**0.5**	90.2% (92)		
No	128 (16.9%)	64.6% (82)	** < 0.001**	**2.1**	86.0% (49)		
Unknown	2 (0.3%)						
Targeted therapy			** < 0.001**			** < 0.001**	
Yes	200 (26.4%)	91.5% (182)	** < 0.001**	**56.2**	96.6% (113)	** < 0.001**	**14.1**
No	556 (73.4%)	14.8% (82)	** < 0.001**	**0.0**	66.7% (28)	** < 0.001**	**0.1**
Unknown	2 (0.3%)						
Initial pathology department			** < 0.001**			**ns**	
Located in expertise center	149 (19.7%)	67.1% (100)	** < 0.001**	**2.4**	96.9% (31)		
Located in tertiary center	56 (7.4%)	67.9% (38)	**0.005**	**2.3**	92.3% (12)		
Located in peripheral center with molecular lab	170 (22.5%)	37.6% (64)	** < 0.001**	**0.5**	78.6% (22)		
Located in peripheral center w/o molecular lab	381 (50.4%)	45.1% (172)	**0.02**	**0.7**	88.4% (76)		
Treatment in expertise center			** < 0.001**			** < 0.001**	
Yes	344 (45.4%)	76.1% (261)	** < 0.001**	**8.5**	96.1% (98)	** < 0.001**	**8.0**
No	414 (54.6%)	27.4% (113)	** < 0.001**	**0.1**	75.4% (43)	** < 0.001**	**0.1**

^†^Predictive analysis includes molecular analysis of patients with high risk or metastatic disease

^††^Mean uptake in the respective group

*Fisher’s exact test was applied to calculate significance

**OR = Odds ratio

^‡^Factors were not included in logistic-regression model as they contribute to the risk group

### Statistical analysis

To identify associations the Fisher’s exact test and multivariate logistic regression were applied using IBM SPSS Statistics (version 25). Obtained odds ratios (ORs) and overall *P*-values (two-sided) are reported. The McNemar test was applied to study paired observations. Overall survival (OS) analyses were performed with R software (version 3.5.3), using the packages cmprsk (version 2.2–9) [35], mstate (version 0.2.12) [36] and survival (version 3.1–8) [37]. Death was counted as event. OS rates were determined using Cox regression and compared using the Wald test.

## Results

### Patient Demographics

**Fig. 2:**
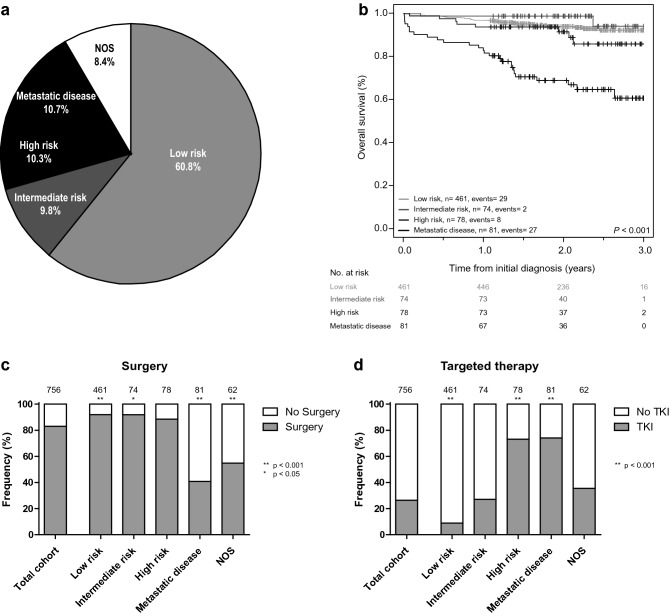
Clinical characteristics of GIST cases diagnosed in the Netherlands in 2017–2018. **a** Cases were classified in risk groups according to the to the AFIP-Miettinen classification [8]. Cases that could not be classified (mostly because the mitotic index was missing) are shown as not otherwise specified (NOS). **b** Overall survival (OS) analysis based on risk groups. OS rates were determined using Cox regression, and compared using the Wald test. The reported *P*-value involves the overall *P*-value. **c**–**d** The frequency of surgery (**c**) and targeted therapy (**d**) in the total cohort and per risk group. The total number of cases that are present in each bar is displayed above the bar. The Fisher’s Exact test was applied to study associations. *TKI* = tyrosine kinase inhibitor. ^**^p < 0.001; ^*^p < 0.05

In 2017 and 2018, 758 patients were diagnosed with a primary GIST in the Netherlands (Fig. 1). The median age of the patients was 67 years, primary tumor localization mainly involved the stomach or small intestine, and the majority of cases was diagnosed with low risk disease (Table 1, Fig. 2a). 21% of the patients showed high risk or metastatic disease and hence were candidates for predictive mutation analysis and targeted therapy. Overall survival (OS) of GIST patients was significantly different between the risk groups (Fig. 2b). Cases with metastatic disease had a shorter OS compared to cases with localized disease (*P* < 0.001). Surgery was predominantly performed in cases with localized disease (Fig. 2c). No details were available of tumor rupture or spill during surgery, which classifies low and intermediate risk cases for adjuvant imatinib therapy. Targeted therapy was registered for 199 cases and was significantly enriched in high risk (OR = 10.2) and metastatic disease cases (OR = 10.9; Fig. 2d).

### Molecular characterization of GIST

**Fig. 3:**
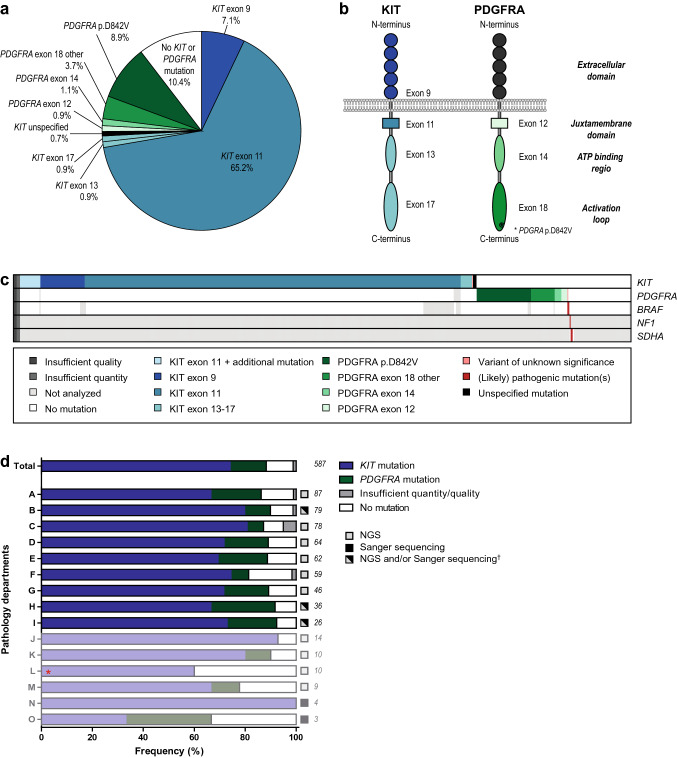
Molecular characteristics of GIST cases and mutation-informed therapeutic choices. **a** Frequency of reported *KIT* and *PDGFRA* mutations. **b** Schematic representation of protein domains of KIT and PDGFRA. **c** Mutational landscape of GIST cases. Each column represents a tumor sample. Each row represents a gene. Tumor samples were sorted on the type of *KIT*/*PDGFRA* mutation. Reported (likely) pathogenic mutations and variants of unknown significance are depicted in the figure. A colored bar represents a variant (see legend), a white bar represents no alteration, and a gray bar represents not analyzed (i.e., not present in NGS panel or single gene analysis of *KIT*/*PDGFRA*). **c** Reported frequencies of *KIT* and *PDGFRA* mutations per pathology department. The technique used for the mutation analyses and number of tests are displayed behind each bar. The diagnostic yield, i.e., the frequency of *KIT* and/or *PDGFRA* mutations, of each pathology department was compared to the diagnostic yield of the remaining departments using the Fisher’s Exact test. *p < 0.05. †Lab B and lab H performed a combination of NGS analysis and Sanger sequencing of *KIT*. Lab I switched from Sanger sequencing to NGS analysis during the data collection

The molecular landscape of 545 GIST patients was studied, involving 374 cases diagnosed in 2017/2018 and 171 cases diagnosed before 2017 (follow-up samples) or after 2018 (Supplementary Fig. 1). For 6 of the 545 cases no results were obtained due to insufficient quality or quantity of the biopsy material. 74.9% of the cases harbored ≥ 1 *KIT* mutation, 14.7% of the cases showed a *PDGFRA* mutation and 10.4% of the cases were *KIT*/*PDGFRA* wildtype (Fig. 3). *PDGFRA* mutations were associated with a poor OS in metastatic disease cases (Supplementary Fig. 2). For the other risk groups, no association between mutations and outcome was observed in the cohort that was linked to the NCR.

A total of 424 *KIT* mutations were reported: 385 cases presented with a single *KIT* mutation, sixteen cases showed two mutations in *KIT* and two cases harbored three *KIT* mutations. These latter two groups consisted of cases harboring secondary resistance mutations (Supplementary Table 2). In addition, 79 cases had a *PDGFRA* mutation, of which the resistance mutation p.D842V (*N* = 48) was most frequent. The distribution of the mutations over the different regions of *KIT* and *PDGFRA* is presented in Fig. 3 and Supplementary Fig. 3a.

Molecular characterization of the 545 GIST patients was performed in 15 (of 38) pathology departments. The number of performed analyses ranged from 3 to 87 analyses per pathology department during 30 months of follow-up. Molecular tests were mainly performed using a targeted NGS-approach whether or not combined with Sanger sequencing of *KIT*. Comparing the total diagnostic yield (i.e., frequency of *KIT* and *PDGFRA* mutations) between the departments showed one department that underperformed the national average diagnostic yield (Fig. 3d). Studying the mutations in the different regions of *KIT* and *PDGFRA* in more detail showed one department that reported a significantly higher frequency of *PDGFRA* mutations and unspecified *KIT* mutations (Supplementary Fig. 3b).

### Molecular testing rates

**Fig. 4:**
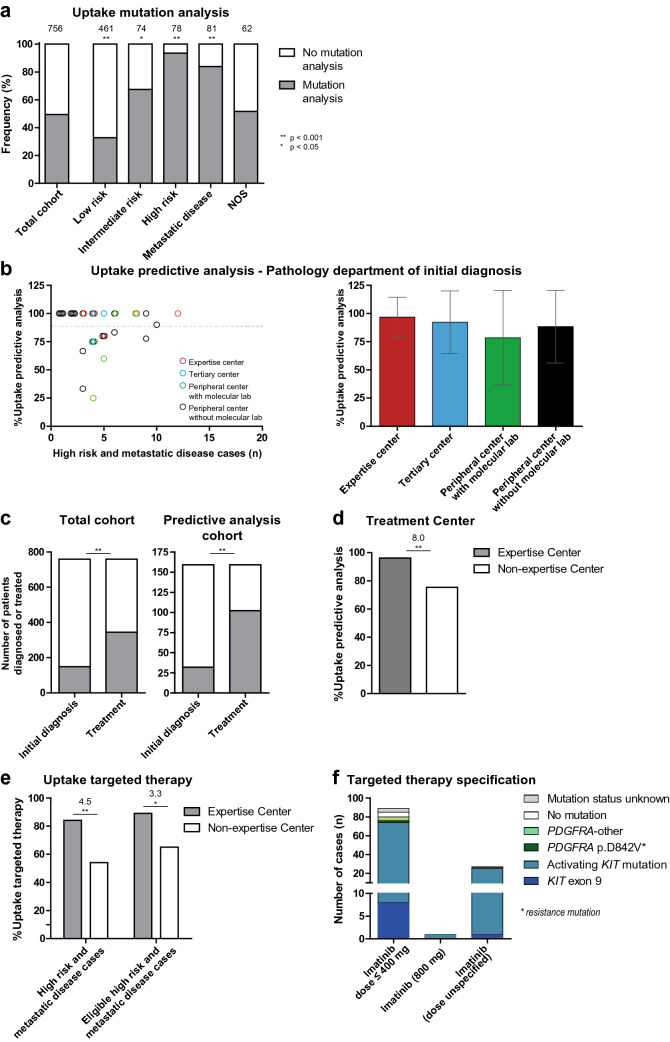
Uptake of predictive molecular analysis and targeted therapy. **a** The frequency of performed mutation analysis in the total cohort and per risk group. The total number of cases that are present in each bar is displayed above the bar. The Fisher’s Exact test was applied to study associations. ^**^p < 0.001; ^*^p < 0.05. **b** Uptake of predictive molecular analysis displayed per pathology department involved in the initial diagnosis. This pathology department could either be located in an expertise center, tertiary cancer center, peripheral center with a molecular laboratory, or a peripheral center without a molecular laboratory, which is displayed by the different colors. The dotted line represents the mean uptake in the total predictive cohort. The bar graph shows the uptake of predictive molecular analysis shown per type of pathology department. Mean ± standard deviation (SD) is shown. Association between the uptake of molecular analysis and the type of pathology laboratory was studied using the Fisher’s exact test. **c** Number of patients initially diagnosed in an expertise or non-expertise center compared to the number of patients treated in an expertise or non-expertise center. Numbers are shown for the total cohort (left) and the predictive analysis cohort (right). The McNemar test was applied to study significance. **d** Uptake of predictive mutation analysis by expertise and non-expertise centers. The Fisher’s Exact test was applied to study differences in uptake. The odds ratio is displayed above the bars. ^**^p < 0.001. **e** Uptake of targeted therapy by expertise and non-expertise centers. Uptake is shown for all cases with high risk or metastatic disease, and for eligible high risk or metastatic disease cases, defined by exclusion of patients that did not receive therapy due to comorbidity, patients’ refusal and/or too high tumor load or presence of the *PDGFRA* p.D842V mutation. The Fisher’s Exact test was applied to study differences in uptake. Odds ratios are displayed above the bars. ^**^p < 0.001; ^*^p < 0.05. **f** Primary therapy specification of 199 GIST cases. Primary therapy includes the therapy that was registered within the first 6–9 months after initial diagnosis. The different types of *KIT/PDGFRA* mutations are shown in different colors

Uptake of mutation analysis was studied in all GIST patients with initial diagnosis in 2017–2018. Molecular testing rates were significantly higher in high risk or metastatic disease cases (89%) compared to low or intermediate risk cases (38%; *P* < 0.01; Fig. 4a; Table 1). Patients were also more likely to receive molecular testing (*P* < 0.001) if they had poorly differentiated tumors, did not undergo surgery, or received targeted therapy. In addition, tumor localization in the rectum or small intestine was associated with a high uptake of mutation analysis (61% and 90%, receptively) compared to localization in the stomach or esophagus (44% and 19%, respectively). The association between clinical variables and uptake of mutation analysis was further analyzed in a multivariate logistic regression model, including risk group, performance status, tumor differentiation, surgery and targeted therapy. Except for surgery and performance status, these factors remained significantly associated with mutation analysis.

Variation in uptake of predictive analysis was studied in patients having an established indication for imatinib therapy, i.e., high risk or metastatic disease cases. These predictive molecular testing rates were independent of the type of pathology department of initial diagnosis (Table 1, Fig. 4b). The center of initial diagnosis is not necessarily the center that requests the molecular analysis and treats the patient, which was emphasized by the higher number of patients treated than initially diagnosed in expertise centers (Fig. 4c). Studying test uptake in the context of treatment center demonstrated a higher uptake of predictive mutation analysis for patients treated in expertise centers (96%) compared to non-expertise centers (75%, *P* < 0.001; Table 1, Fig. 4d). This association remained significant in a multivariate logistic-regression model including risk group, tumor differentiation, targeted therapy, and a variable indicating exclusion from further therapy (e.g. comorbidities, poor performance status or patients’ choice).

### Mutation-tailored targeted therapy choices

Variation in uptake of imatinib therapy and mutation-tailored therapeutic choices, as recommended by the ESMO guidelines [19, 20], was also studied in these high risk or metastatic disease cases. 117 (74%) of these 159 cases received imatinib therapy. Uptake of imatinib therapy was significantly higher in expertise centers (84%) compared to non-expertise centers (54%, *P* < 0.001, OR = 4.5) (Fig. 4e). 11% (17/159) of the cases with high risk or metastatic disease were not eligible for imatinib therapy due to comorbidity, patients’ refusal, too high tumor load or presence of the *PDGFRA* p.D842V imatinib-resistance mutation. After exclusion of these cases, 81% (115/142) of the remaining cases were treated with imatinib therapy, which remained significantly higher in the expertise centers (89%) compared to non-expertise centers (65%; *P* = 0.002, OR = 4.1). Two untreated cases were *KIT* and *PDGFRA* wildtype, while for 25 cases it remained unclear why they did not receive imatinib therapy. Eight of these cases did not undergo molecular testing, 16 cases harbored a *KIT* mutation, one case a sensitizing *PDGFRA* mutation.

Two of the five patients that were diagnosed with high risk or metastatic disease and the primary resistance mutation *PDGFRA* p.D842V received imatinib therapy (Fig. 4f). Treatment was given in one expertise and one non-expertise center. Nine of twelve cases diagnosed with the *KIT* exon 9 mutation received imatinib therapy, which involved the normal dose in eight of the patients and an unspecified dose in one patient. Four patients received imatinib therapy while no molecular analysis was performed.

In summary, 81% (115/142) of the eligible high risk or metastatic disease patients received imatinib therapy. 2% (2/117) of the imatinib-treated patients received therapy despite the *PDGFRA* p.D842V resistance mutation, 7% (8/117) of the patients initiated imatinib therapy at the normal instead of high dose in spite of having a KIT exon 9 mutation, and 3% (4/117) of the imatinib-treated patients were not molecularly characterized. Taken together, 73% (103/142) of the eligible high risk or metastatic disease patients received mutation-tailored therapy according to the ESMO guideline.

## Discussion

Evaluation of mutation-informed treatment of GIST demonstrated that over 80% of the GIST patients with high risk or metastatic disease are molecularly tested and treated with imatinib mostly in line with ESMO guidelines [19, 20]. Overall, we showed that 89% of the GIST patients with high risk or metastatic disease underwent predictive testing. This uptake was independent of the center performing the initial diagnosis, which was not necessarily the center that requested the molecular test and treated the patient. In contrast, this predictive analysis was more often performed for patients treated in expertise centers compared to non-expertise centers. Likewise, uptake of imatinib therapy was higher for patients with high risk or metastatic disease that were treated in expertise centers, suggesting that treatment in expertise centers improves the therapeutic management of GIST patients. In general, 81% of the patients with high risk or metastatic disease, without adverse characteristics like comorbidities, too high tumor load or the *PDGFRA *p.D842V resistance mutation, received imatinib therapy. These results are in line with the recent study of Nishida et al. [30]. Molecular analysis preceded imatinib therapy in 97% of the cases. For the cases with the *PDGFRA* p.D842V resistance mutation that received imatinib therapy, it remains unclear whether therapy was initiated due to insufficient knowledge or whether the test result was not noticed by the treating clinician. Primary therapy for patients that harbor *KIT* exon 9 mutations mainly involved the standard dose of imatinib, instead of the high dose as advised by the ESMO guideline [19, 20]. A possible explanation might be that therapy was initiated with this standard imatinib dose to prevent more severe side effects that are observed upon treatment with the high imatinib dose [38] and was increased upon progressive disease. However, as only the primary systemic therapy was registered by the NCR, we could not evaluate whether treatment dose was adjusted over time. Taken together, these observations show that performing predictive mutation analysis does not guarantee mutation-tailored treatment selection and that one in four eligible patients was not treated according to the recommendations in the guideline.

Although this study focused on uptake of predictive analysis and mutation-informed therapy in patients with high risk or metastatic disease, also data regarding uptake of mutation analysis in low and intermediate risk patients were obtained. Only a minority of these patients underwent molecular characterization instead of all patients as proposed by the guidelines [19, 20]. These low molecular testing rates suggest that a selective approach is often used for mutation analysis, which focusses on GIST patients that are candidates for imatinib therapy. This approach is likely used to reduce cost burden of diagnostic procedures. As the additive value of testing low and intermediate risk patients is limited, it may be justifiable not to test these patients. The observations presented in the current study should be used to consider whether guidelines adjustments should be made based on actual practice.

An overview of the molecular landscape of GIST cases showed *KIT* mutations in 74.8% of the cases, *PDGFRA* mutations in 14.8% of the cases, and 10.4% of the cases were *KIT*/*PDGFRA* wildtype, which was in line with other studies [15, 39–45]. Although numbers were limited, patients with metastatic disease and *PDGFRA* mutations had a poor OS compared to the remaining metastatic disease patients. This is likely explained by absence of or a poor response to targeted therapy [21, 46, 47]. In contrast, in literature, it has been suggested that localized GISTs with *PDGFRA* mutations are associated with more favorable prognosis [48–50]. Due to limitations in the data collection strategy, we could not analyze the association between *PDGFRA* mutations and recurrence in the current study. However, we did not observe an aberrant OS of *PDGFRA* mutated GIST patients in localized disease.

The quality of mutation testing is essential for the diagnosis and treatment of GIST patients and therefore performance of the pathology departments was studied. Variant detection was performed by 15 individual pathology departments. One pathology department underperformed the national average diagnostic yield, whereas the remaining 14 departments showed comparable overall mutation frequencies of *KIT* and *PDGFRA*. However, power for comparison of mutational frequencies was limited, as 9/15 laboratories performed less than 50 analyses during the inclusion period of 2.5 years. These low number also affected the power to compare the frequency of the different mutation types between the pathology departments. Nevertheless, one department was identified that outperformed the national average of *PDGFRA* mutations.

Our study was limited to the data collection design of the two registries. Tumor rupture or spill during surgery, an important prognostic variable, was not registered by the NCR. Hence, uptake of predictive analysis and targeted therapy could not be studied in low and intermediate risk cases. However, by limiting the analysis of uptake to those patients with an established indication for predictive testing and imatinib therapy, this did not affect our results. As clinical variables were registered only once, i.e., 6–9 months after initial diagnosis, we were unable to study changes in therapy (dose) and relapses. This did not affect or explain the main result of suboptimal uptake of testing and subsequent targeted therapy.

In conclusion, nationwide real-world data show that over 80% of the patients with high risk or metastatic disease receive predictive analysis and targeted therapy. Predictive analysis did not guarantee treatment according to ESMO guideline as only 91% actually received mutation-tailored treatment. Therefore, one in four patients that could opt for targeted treatment were not treated according to the recommendations in the guideline. The reasons for suboptimal uptake of testing and treatment require further study.

## Supplementary Information

Below is the link to the electronic supplementary material.

Supplementary file1 (PDF 175 KB)

Supplementary file2 (XLSX 116 KB)

## Data Availability

The data that support the findings of this study are available upon reasonable request from the corresponding author. The data are not publicly available due to privacy or ethical restrictions.
