# Supplementary material for: Nationwide evaluation of mutation-tailored treatment of gastrointestinal stromal tumors in daily clinical practice
Source: Gastric Cancer. 2021 Apr 28;24(5):990–1002. doi: 10.1007/s10120-021-01190-9 (PMC8338807; doi:10.1007/s10120-021-01190-9)
Supplement: Supplementary file 1 — Supplementary file1 (PDF 175 KB) [file 10120_2021_1190_MOESM1_ESM.pdf]

Supplementary Figure 1

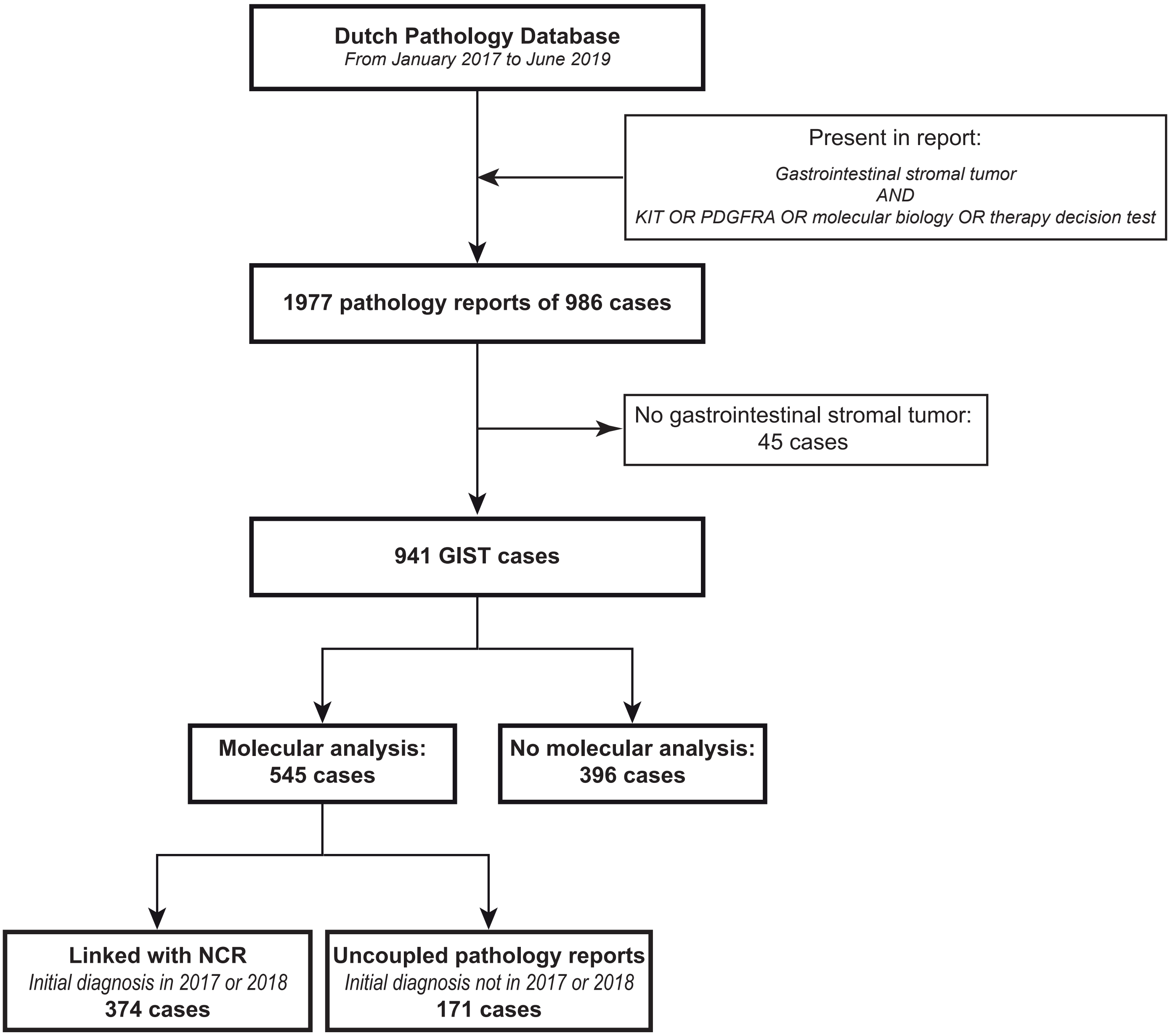

Supplementary Figure 1: Flow chart of the data collection from the Dutch Pathology Register
Pathology reports were collected from the Dutch Pathology Register (PALGA) from January 2017 till June 2019. Reports were retrieved matching the following search criteria: gastrointestinal stromal tumor AND *KIT* OR *PDGFRA* OR molecular biology OR therapy decision test. Reports that were collected based on the search criteria were manually curated based on the presence of mutation analysis.

Supplementary Figure 2

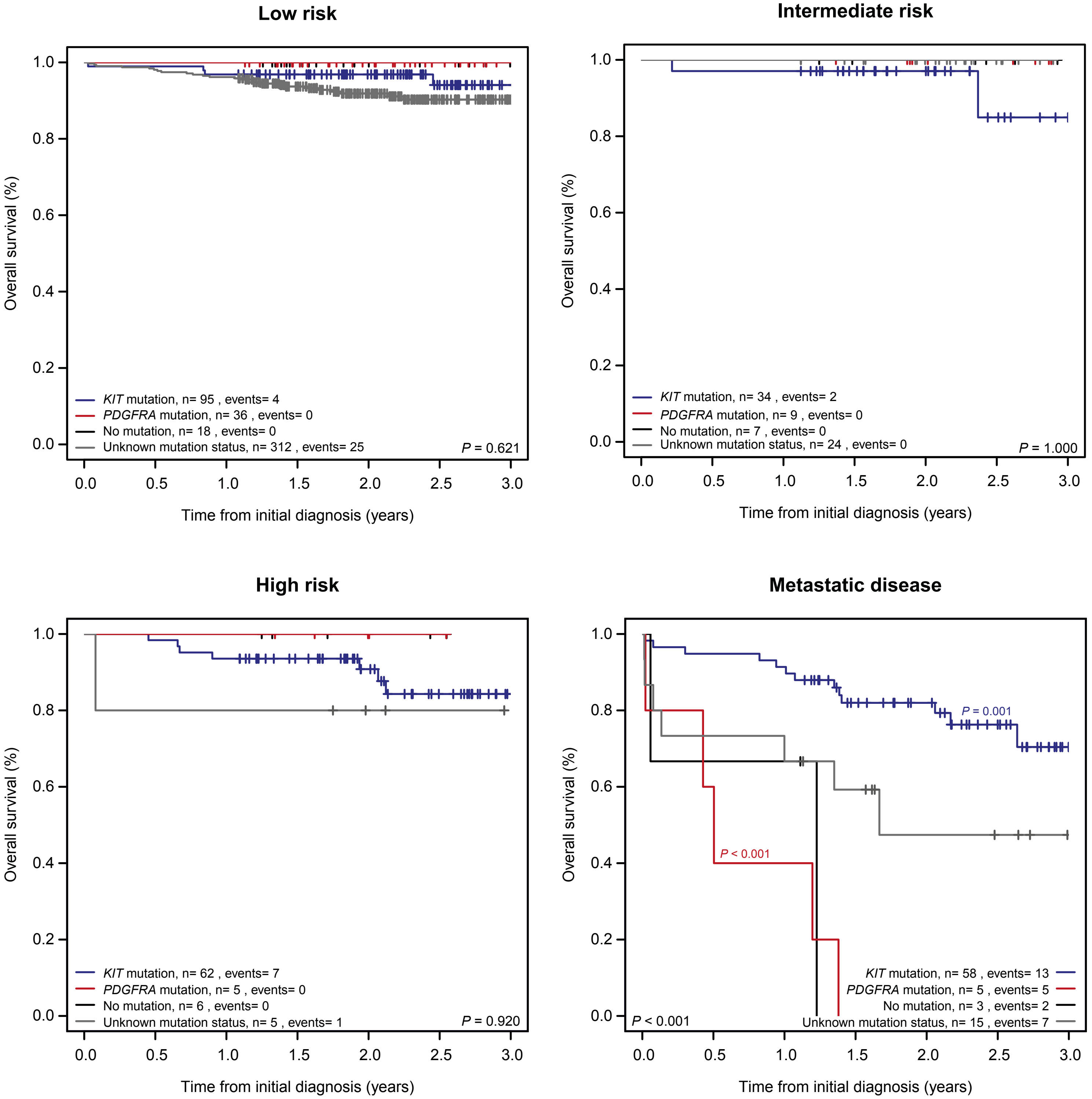

Supplementary Figure 2: Overall survival of GIST cases based on mutation status
Overall survival (OS) of GIST cases based on the mutation status. Analyses were performed per disease stage. OS rates were determined using Cox regression and compared using the Wald test. Overal p-values are reported within each graph. In case of significance, each individual group was compared to the remaing patients. Imatinib therapy (yes/no) was included as covariate in these models. Signficant p-values for these individual groups are shown.

Supplementary Figure 3

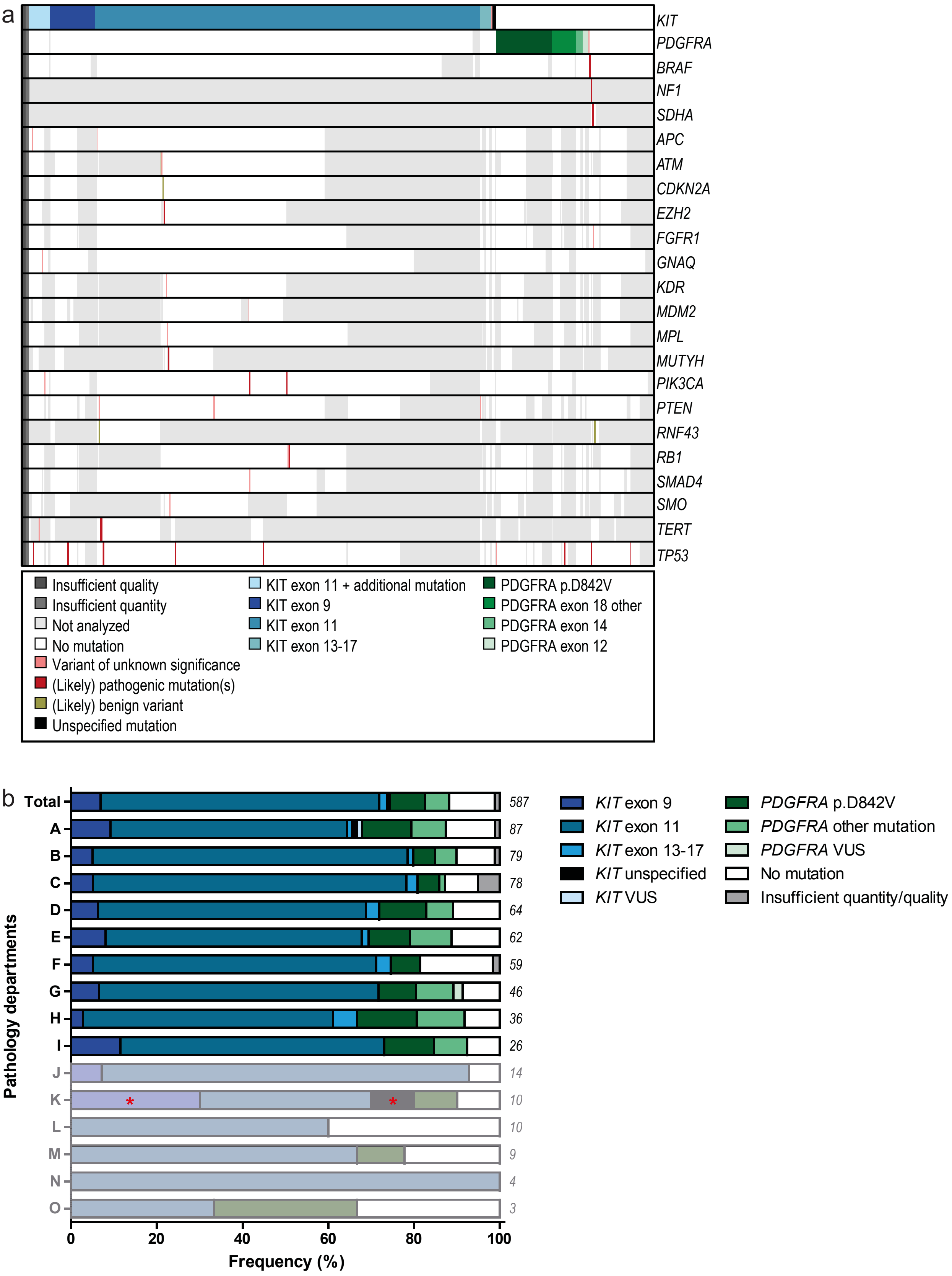

Supplementary Figure 3: Molecular diagnostic analyses of GIST cases.
(**a**) Mutational landscape of GIST cases. Each column represents a tumor sample. Each row represents a gene. Tumor samples were sorted on the type of KIT/PDGFRA mutation. Reported (likely) pathogenic mutations, variants of unknown significance and (likely) benign variants are depicted in the figure. A colored bar represents a variant (see legend), a white bar represents no alteration, and a grey bar represents not analyzed (i.e. not present in NGS panel or single gene analysis of KIT/PDGFRA). (**b**) Reported frequencies of *KIT* and *PDGFRA* mutations per pathology department. The number of tests per bar are displayed behind each bar. Frequencies per pathology departments were compared the respective frequencies of the remaining pathology departments using the Fisher's Exact test. * p<0.05
